# Mitral annular disjunction in myxomatous mitral valve disease: a relevant abnormality recognizable by transthoracic echocardiography

**DOI:** 10.1186/1476-7120-8-53

**Published:** 2010-12-09

**Authors:** Pedro Carmo, Maria J Andrade, Carlos Aguiar, Rui Rodrigues, Raquel Gouveia, José A Silva

**Affiliations:** 1 Cardiology Department, Hospital de Santa Cruz, Avenida Prof. Reinaldo dos Santos, 2790-134 Carnaxide, Portugal; 2 Cardiothoracic Surgery Department, Hospital de Santa Cruz, Avenida Prof. Reinaldo dos Santos, 2790-134 Carnaxide, Portugal

## Abstract

**Background:**

Mitral annular disjunction (MAD) consists of an altered spatial relation between the left atrial wall, the attachment of the mitral leaflets, and the top of the left ventricular (LV) free wall, manifested as a wide separation between the atrial wall-mitral valve junction and the top of the LV free wall. Originally described in association with myxomatous mitral valve disease, this abnormality was recently revisited by a surgical group that pointed its relevance for mitral valve reparability. The aims of this study were to investigate the echocardiographic prevalence of mitral annular disjunction in patients with myxomatous mitral valve disease, and to characterize the clinical profile and echocardiographic features of these patients.

**Methods:**

We evaluated 38 patients with myxomatous mitral valve disease (mean age 57 ± 15 years; 18 females) and used standard transthoracic echocardiography for measuring the MAD. Mitral annular function, assessed by end-diastolic and end-systolic annular diameters, was compared between patients with and without MAD. We compared the incidence of arrhythmias in a subset of 21 patients studied with 24-hour Holter monitoring.

**Results:**

MAD was present in 21 (55%) patients (mean length: 7.4 ± 8.7 mm), and was more common in women (61% vs 38% in men; p = 0.047). MAD patients more frequently presented chest pain (43% vs 12% in the absence of MAD; p = 0.07). Mitral annular function was significantly impaired in patients with MAD in whom the mitral annular diameter was paradoxically larger in systole than in diastole: the diastolic-to-systolic mitral annular diameter difference was -4,6 ± 4,7 mm in these patients vs 3,4 ± 1,1 mm in those without MAD (p < 0.001). The severity of MAD significantly correlated with the occurrence of non-sustained ventricular tachycardia (NSVT) on Holter monitoring: MAD›8.5 mm was a strong predictor for (NSVT), (area under ROC curve = 0.74 (95% CI, 0.5-0.9); sensitivity 67%, specificity 83%). There were no differences between groups regarding functional class, severity of mitral regurgitation, LV volumes, and LV systolic function.

**Conclusions:**

MAD is a common finding in myxomatous mitral valve disease patients, easily recognizable by transthoracic echocardiography. It is more prevalent in women and often associated with chest pain. MAD significantly disturbs mitral annular function and when severe predicts the occurrence of NSVT.

## Introduction

**Figure 1:**
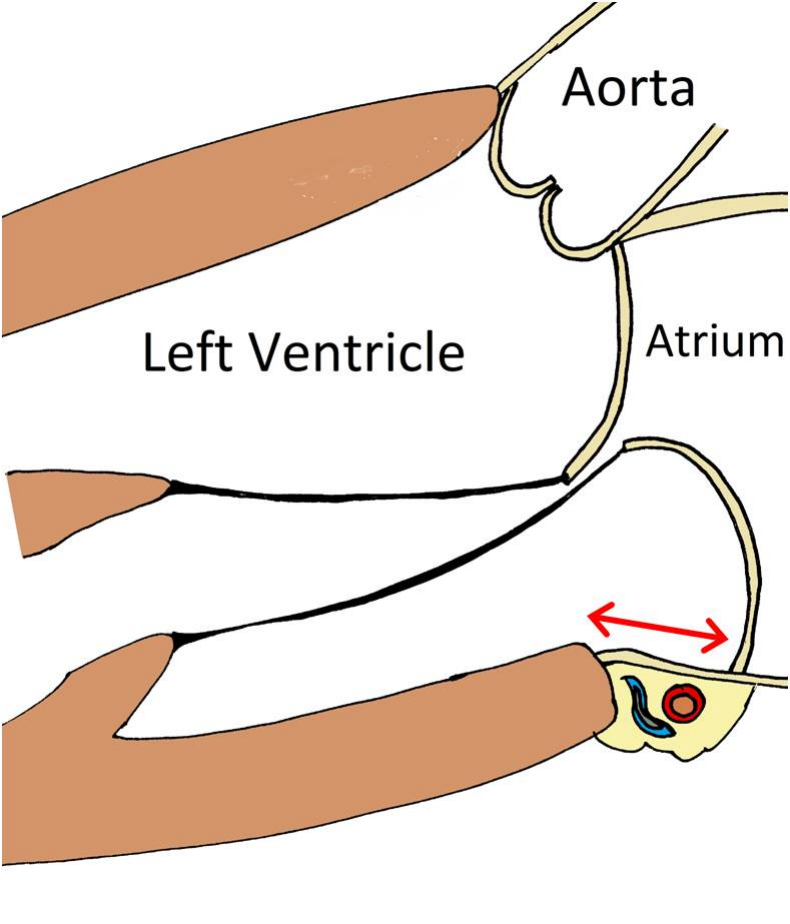
**Schematic representation of mitral annular disjunction (arrow)**.

**Figure 2:**
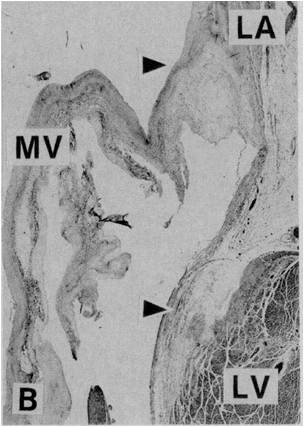
**Histological section from the region of the mitral annulus showing a wide separation between the insertion of mitral valve and the left ventricular top border (arrowheads)**. Reproduced with permission from Hutchins GM, Moore GW, Skoog DK. "The association of floppy mitral valve with disjunction of the mitral annulus fibrosus". N Engl J Med 1986; 314: 535-540.

**Figure 3:**
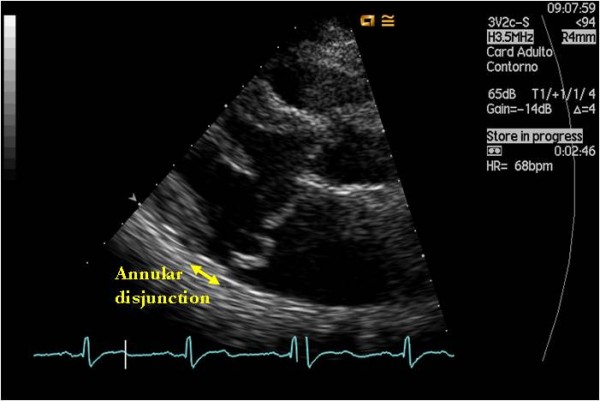
**Parasternal long axis view of a myxomatous mitral valve showing significant disjunction of the posterior annulus**.

Mitral annular disjunction consists of a perceptible separation between the left atrial wall-mitral valve junction and the top of the left ventricle wall (Figures 1, 2 and 3.). Originally described more than 20 years ago by Hutchins et al [1], these authors found a strong association between floppy mitral valve and mitral annular disjunction. They further suggested that the disjunction of the mitral annulus fibrosus could play a role in the development of the pathological features of myxomatous valve disease through the mechanical stress incited by the excessive mobility of the mitral apparatus [1].

This abnormality was forgotten until a Canadian surgical group recently stated the relevance of its recognition prior to mitral valve repair [2]. In these patients, a modification of the surgical technique seems necessary to avoid prosthetic valve replacement, and to guarantee an optimal and long-lasting result of the repair. Aside these surgical considerations, mitral annulus disjunction has received little attention, and the clinical and transthoracic echocardiographic characteristics of these patients are largely unknown.

For a long time, at our echo laboratory, we have been confronted with the recognition of this structural abnormality which we have empirically associated with distinct clinical features, namely a high incidence of arrhythmias.

The aims of our study were to determine the prevalence of echocardiographically-recognized mitral annular disjunction in patients with myxomatous valve disease, and to compare the clinical profile and echocardiographic features of patients with and without this abnormality.

## Methods

### Study population

For the purpose of this study, we reassessed the clinical and echocardiographic data from all patients with myxomatous mitral valve disease who underwent a transthoracic echocardiographic examination in our laboratory between July 2003 and September 2006. Myxomatous mitral valve was defined as the presence of excess leaflet tissue and leaflet thickening greater than 5 mm, resulting in a prolapse greater than 2 mm into the left atrium on parasternal long axis view [3]. Overall, 38 patients were included and there were no exclusion criteria.

### Echocardiographic examination

#### Comprehensive two-dimensional Doppler echocardiography

All patients underwent a complete transthoracic 2D, M-mode, and Doppler examination using commercially available systems (Powervision 7000, Toshiba; Acuson Sequoia 320, Siemens; Vivid 7, General Electrics). Image acquisitions and measurements were carried out by senior echocardiographers in accordance with the *European Association of Echocardiography *recommendations [4]. The left ventricle was evaluated using left ventricular end-diastolic and end-systolic diameters (LVEDD, LVESD), fractional shortening (FS), LV end-diastolic and end-systolic volumes (LVEDV, LVESV), and ejection fraction (EF) from the modified biplane Simpson's method. Left atrial (LA) size was measured by M-mode. Pulmonary artery systolic pressure (PASP) was estimated from the tricuspid regurgitation jet peak velocity according to the modified Bernoulli equation. Mitral regurgitation (MR) severity was evaluated by colour Doppler combined with the width of the vena contracta and the calculation of the effective regurgitant orifice area by the proximal flow convergence (PISA) method whenever feasible. MR severity was graded according to the *European Association of Echocardiography *recommendations [5].

#### Detection and measurement of mitral annular disjunction and annular diameters

The length of annular disjunction was measured from the left atrial wall-mitral valve posterior leaflet junction to the top of the LV posterior wall during end-systole (Figures 1 and 3). Mitral annular function was evaluated by measuring the mitral annular diameter during end-systole and end-diastole, on a parasternal long axis view. The difference between these two measurements was considered positive whenever the end-systolic diameter was smaller than the end-diastolic diameter, as is usually seen in normal mitral valve kinetics.

### 24-Hour Holter monitoring

In order to evaluate the arrhythmic profile, a subset of 21 patients not submitted to mitral valve surgery was further studied with 24-hour Holter monitoring. The frequency of ventricular tachycardia was quantified by the sum of beats in ventricular tachycardia.

### Statistical analysis

All data presented are shown as mean ± standard deviation or absolute number (percentage). To compare the differences between groups, we used Student's *t *test for continuous data and the chi-square test or Fisher's exact test for categorical data, as appropriate. The Mann-Whitney test was used to compare differences between continuous non-parametric variables. The ANOVA method was used to compare differences between more than two groups. Receiver Operating Characteristic (ROC) curve analysis was performed to determine the disjunction length that more accurately predicted the occurrence of non-sustained ventricular tachycardia. Inter and intra-observer correlations were evaluated by Pearson correlation coefficient analysis. Statistical significance was accepted for a two-tailed P < 0.05. SPSS for Windows version 13 (SPSS Inc, Chicago, Ill) and MedCalc for Windows version 9.3.8.0 (MedCalc Software, Mariakerke, Belgium) were used to perform the statistical analyses.

## Results

### Baseline characteristics

Clinical and echocardiographic characteristics of the population are shown in tables 1 and 2, respectively. The majority of patients were symptomatic with a mean NYHA class of 1.3 ± 0.9. Three patients (8%) had a NYHA class greater than 2. Every patient had some degree of mitral valve regurgitation, which was severe in 25 (66%). Eleven patients had already undergone mitral valve surgery (valve repair in four, valve replacement in five, and valve repair followed by valve replacement in two).

**Table 1:** Baseline clinical characteristics

Variables	Total n = 38	With Disjunction n = 21	Without Disjunction n = 17	P
Mean age, years	58 ± 15	60 ± 15	55 ± 15	Ns
Female sex, n(%)	18 (47)	13 (62)	5 (29)	0.047
Mean age at diagnosis, years	46 ± 21	50 ± 19	41 ± 22	Ns
Coronary arterial disease, n(%)	1 (3)	1 (5)	0 (0)	Ns
Chronic atrial fibrillation, n(%)	6 (16)	3 (14)	3 (18)	Ns
Paroxysmal atrial fibrillation, n(%)	4 (11)	3 (14)	1 (6)	Ns
Stroke, n(%)	3 (8)	3 (14)	0 (0)	Ns
Endocarditis, n(%)	2 (5)	0 (0)	2 (12)	Ns
Symptomatic, n(%)	35 (92)	21 (100)	14 (82)	0.08
NYHA class, I/II/III/IV, n(%)	20(53)/15(39)/3(8)/0(0)	11(52)/8(38)/2(10)/0(0)	9(53)/7(41)/1(6)/0(0)	Ns
Mean NYHA class	1.3 ± 0.9	1.4 ± 0.9	1.2 ± 1.0	Ns
Syncope, n(%)	5 (13)	2 (10)	3 (18)	Ns
Palpitations, n(%)	12 (32)	6 (29)	6 (35)	Ns
Chest Pain, n(%)	11 (29)	9 (43)	2 (12)	0.07
Mitral repair surgery only, n(%)	4 (11)	1 (5)	3 (18)	Ns
Mitral prosthesis only, n(%)	5 (13)	3 (14)	2 (12)	Ns
Mitral repair and replacement, n(%)	2 (5)	1 (5)	1 (6)	ns

**Table 2:** Echocardiographic Parameters

Variables	Total n = 38	With Disjunction N = 21	Without Disjunction n = 17	P
EDD, mm	57 ± 9	56 ± 10	58 ± 8	ns
EDV, mL	155 ± 41	141 ± 72	169 ± 48	ns
EF, %	66 ± 7	66 ± 7	67 ± 7	ns
FS, %	40 ± 6	40 ± 5	38 ± 6	ns
LA, mm	47 ± 11	45 ± 10	49 ± 12	ns
PASP, mmHg	34 ± 12	38 ± 7	30 ± 14	ns
MR, I, II, III, n (%)	5 (13)/8(21)/25(66)	2(9)/6(29)/13(62)	3(18)/2(12)/12(70)	ns
EROA, mm2	38 ± 20	33 ± 21	42 ± 19	ns
Mitral Annular diameter (diastolic-systolic), mm	-1.1 ± 5.4	-4.6 ± 4.7	3.4 ± 1.1	< 0.001

EDD: Left ventricular end-diastolic diameter; EDV: Left ventricular end-diastolic volume; FS: fractional shortening; EF: ejection fraction; LA: left atrial diameter; PASP: Pulmonary artery systolic pressure; MR: mitral regurgitation; I: mild; II: moderate; III: severe; EROA: effective regurgitant orifice area

Mitral annular disjunction was seen in 21 (55%) patients and on average measured 7.4 ± 8.7 mm. Inter and intra-observer correlations were 0.97 and 0.94, respectively. The most severely affected patient had a disjunction length of 30 mm. This particular patient had involvement of the entire annular circumference, and this feature was well documented by transoesophageal echocardiography (Additional file 1). Patients with mitral annular disjunction were more often females (62% vs 38%; p = 0.047). Chest pain showed a trend to be more prevalent among patients with than without mitral annulus disjunction (43% vs 12%; p = 0.07). There were no differences between groups regarding NYHA functional class, mitral regurgitation severity or left ventricular ejection fraction (Tables 1 and 2). The transthoracic features of this abnormality are shown in Additional files 2, 3 and 4.

### Mitral annulus function

**Figure 4:**
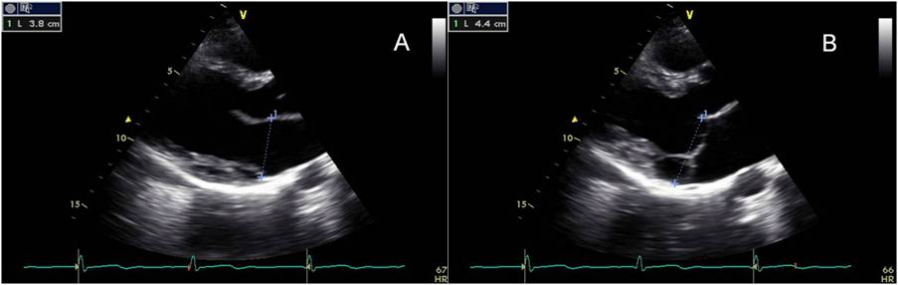
**Annular diameter at end-diastole (A) and end-systole (B) in a patient with annular disjunction. There is a paradoxical increase of the diameter during systole**.

We found an association between the presence of disjunction and mitral annulus dysfunction (Table 2). In the disjunction group, we observed a paradoxical increase of the mitral annulus diameter during systole. The diastolic-to-systolic mitral annulus diameter difference was -4.6 ± 4.7 mm in this group vs 3.4 ± 1.1 mm in the group without mitral annular disjunction (p < 0.001) (Figure 4 and Additional file 5).

### Arrhythmic profile

**Figure 5:**
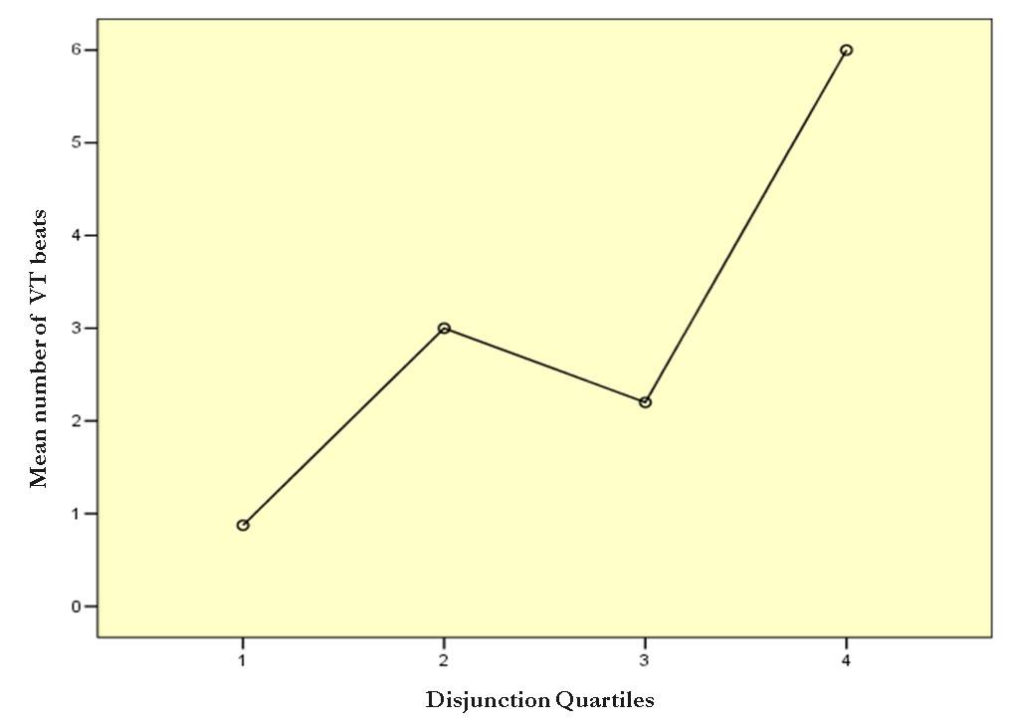
**Mean number of VT beats distributed according to quartiles of disjunction length**.

We observed a high prevalence of atrial fibrillation in the study population. Six patients (16%) were in permanent atrial fibrillation, and another 4 patients (11%) had at least one episode of paroxysmal atrial fibrillation. There were no differences regarding atrial fibrillation frequency between the group with and without annular disjunction (Table 1). A subset of 21 patients not submitted to mitral valve surgery was further studied with 24-hour Holter monitoring (Table 3). There was no record of sustained ventricular arrhythmia during Holter monitoring. The group with annular disjunction had an increased frequency of ventricular extra beats and non-sustained ventricular tachycardia (NSVT), though this relation wasn't statistically significant. Nevertheless, we found that the wider the magnitude of the disjunction, the higher the incidence of NSVT (Figure 5). A disjunction greater than 8.5 mm was a reasonable criterion to predict the risk of NSVT with a sensitivity of 67% and a specificity of 83% (Odds ratio = 10; 95% CI: 1.28 -78.1).

**Table 3:** Holter monitoring Profile

Variables	Total n = 21	With Disjunction N = 21	Without Disjunction n = 17	P
Ventricular extra beats, median (interquartile range)	113 (29; 580)	145 (26; 955)	102 (31; 551)	ns
Supraventricular extra beats, median (interquartile range)	28 (9; 198)	27 (6; 172)	37 (13; 622)	ns
Beats in ventricular tachycardia, mean ± standard deviation	3 ± 5	3 ± 5	1 ± 2	ns
Beats in supraventricular tachycardia, median (interquartile range)	3 (0; 6)	3 (0; 6)	4 (0; 6)	ns

## Discussion

Mitral annular disjunction has been scarcely mentioned in the literature. In a paper based on a review of 900 random histological mitral annulus exams at necropsy, Hutchins et al [1] describes a wide range of normal anatomic variation for this region. These authors observed mitral annulus disjunction in 65 (7%) hearts, 23 of them in association with floppy mitral valve. This abnormality was also seen in association with isolated calcified mitral annulus, and with otherwise normal hearts. Because patients with isolated annular disjunction were younger than those with associated floppy mitral valve it was suggested that the disjunction could play a role in the pathogenesis of myxomatous valve disease, through the increased mechanical stress induced by the excessive mobility of the mitral leaflets [1].

As for our results, the prevalence of annular disjunction in the setting of myxomatous valve disease is significantly high (55%). Although impressive, this proportion is nevertheless smaller than the 92% prevalence found by Hutchins et al [1], and the 98% prevalence found by Eriksson et al [2] with transoesophageal echocardiography in patients with advanced forms of myxomatous mitral valve disease. Both the reduced sensitivity of a transthoracic examination and the use of different diagnostic criteria may account for these discrepancies.

Real-time three-dimensional (3D) and 3D reconstruction transoesophageal echocardiography affords a better accuracy in patients with complex MV pathology when compared with 2D transoesophageal echocardiography.[6,7] The complex structure of the mitral annulus makes it particularly suited to 3D assessment. MV may be viewed from either atrial or ventricular perspectives (en face) which permit a full length view of the mitral annulus contrasting with the segmental view offered by 2D assessment. This feature can enhance the sensitivity for the detection of mitral annular disjunction. However, the image quality using transthoracic echocardiography is sometimes poor weakening its advantage.

To the best of our knowledge, this is the first study where the recognition of mitral annular disjunction is described by transthoracic echocardiography. On intraoperative transoesophageal echocardiography, Eriksson et al described a significantly higher rate of mitral annular disjunction in patients with advanced versus mild or moderate mitral valve degeneration (98% vs 9%)[2]. In our series, there wasn't any relation between annular disjunction and other specific echocardiographic features, namely the degree of mitral valve regurgitation, atrial or ventricular enlargement and ventricular function.

Mitral annulus contractility contributes significantly to mitral valve function. Shortening of the annulus diameter during systole facilitates coaptation of the mitral leaflets [8-10]. Impairment of mitral annulus function is known to be associated with mitral regurgitation associated with myxomatous mitral valve disease, and has recently been implicated as a cause for valve repair failure [10,11].

In the presence of annular disjunction, the valve insertion in the "atrial wall" is responsible for an increased diameter of the mitral valve circumference during systole, and hence impaired annular function due to coaptation deficit. Underestimating this abnormality during mitral valve repair can result in recurrent mitral regurgitation, since paradoxical systolic enlargement will persist [2].

The risk of sudden death is increased in patients with mitral regurgitation due to myxomatous mitral valve disease. Prior to surgery, the incidence of sudden death is 1.8% per year, accounting for one-fourth of the causes of death. Patients with severe symptoms, atrial fibrillation, and reduced LV systolic function are at higher risk. However, even asymptomatic patients in sinus rhythm with normal LV function are not exempt of risk, which occurs with an incidence of 0.8% per year [12-15]. Increased frequency of ventricular arrhythmias in myxomatous mitral valve disease may result from abnormal excessive traction on papillary muscles, generated by the parachuting closure of the mitral valve [16]. As proposed by Hutchins et al, the annular disjunction may, for itself, increase the tension over the mitral apparatus [1]. This mechanism could hypothetically predispose to ventricular arrhythmias. In our study, despite the absence of sustained ventricular arrhythmias, there was an increased frequency of ventricular extra beats and of non-sustained ventricular tachycardia in patients with greater lengths of annular disjunction.

The limitations of this study are its retrospective nature, the rather small population, and the performance of 24-hour Holter monitoring in a limited subset of patients. Larger and prospective studies are needed to validate our findings. Nevertheless, the novelty of the subject and the implications for both surgical management and for arrhythmic events remain of paramount importance.

## Conclusions

Mitral annular disjunction is a common finding in patients with myxomatous mitral valve disease, easily detected and measured by transthoracic echocardiography.

We found an association between this abnormality and several clinical features namely chest pain, annular contractile dysfunction, and non-sustained ventricular tachycardia in cases of wider magnitude of the disjunction. It proved to be relevant for the success of mitral valve repair.

Further and larger studies are needed to completely understand the clinical implications of annular disjunction, particularly its association with malignant arrhythmic events.

## Abbreviations

MAD: Mitral annular disjunction; LV: Left ventricular; NSVT: Non-sustained ventricular tachycardia;

## Competing interests

The authors declare that they have no competing interests.

## Authors' contributions

PC and MJA were responsible for study design, analysis and interpretation of data, manuscript drafting and critical revision. CA contributed to analysis and revised the manuscript for important intellectual content. RR contributed to study design. RG and JAS have supervised and commented the manuscript. All authors read and approved the final manuscript.

## Supplementary Material

Additional file 1 **Movie 1**. Disjunction affecting the entire mitral annular circumference in a transoesophageal echocardiogram. Click here for file

Additional file 2 **Movie 2**. Parasternal long axis view showing significant disjunction of the posterior annulus. Click here for file

Additional file 3 **Movie 3**. Apical 4-chambers view showing disjunction of the lateral annulus. Click here for file

Additional file 4 **Movie 4**. Apical 2-chambers view showing wide disjunction of anterior and inferior mitral annulus. Click here for file

Additional file 5 **Movie 5**. Significant mitral disjunction of the posterior annulus (top left), and of the inferior annulus (top right). Paradoxical annulus enlargement during systole (Bottom). Click here for file
